# Prognostic Significance of NOTCH1-ICD Expression in Renal Cell Carcinoma

**DOI:** 10.15586/jkc.v13i2.446

**Published:** 2026-05-08

**Authors:** Milica Višić, Justine Paris, Manh Duc Hoang, Duško Dunđerović, Snežana Živković Perišić, Sanja Radojević Škodrić, Guilhem Bousquet, Jelena Filipović

**Affiliations:** 1 Faculty of Medicine, University of Belgrade, 11000 Belgrade, Serbia;; 2 Université Sorbonne Paris Nord, 93430 Villetaneuse, France; Service d’Oncologie Médicale, AP-HP, Hôpital Avicenne, 93000 Bobigny, France; 3 National Cancer Hospital, Cancer Research and Clinical Trials Center, Ha Noi, Vietnam;; 4 Institute of Pathology, Faculty of Medicine, University of Belgrade, 11000 Belgrade, Serbia;; 5 Institute of Public Health “Dr. Milan Jovanović Batut,” 11000 Belgrade, Serbia

**Keywords:** Renal cell carcinoma, NOTCH1-ICD, Clinical outcome, Targeted therapy

## Abstract

The NOTCH1 signaling pathway regulates proliferation, differentiation, and apoptosis, with its intracellular domain (NOTCH1-ICD) reflecting pathway activation. While NOTCH1 dysregulation has been linked to renal cell carcinoma (RCC), its prognostic significance across RCC subtypes remains unclear. In this study, we analyzed NOTCH1-ICD immunohistochemical expression in 101 RCC patients: 69 clear cell RCC (ccRCC), 15 papillary RCC (pRCC), and 17 chromophobe RCC (chRCC), and correlated results with clinicopathological features and survival. In ccRCC, high NOTCH1-ICD expression (>15% positive nuclei) identified a small subgroup of tumors with aggressive features and a trend toward poorer overall survival; however, in multivariate analysis, tumor grade emerged as the only independent prognostic factor (HR = 3.36, 95% CI: 1.07–10.49, p = 0.037), while NOTCH1 showed nonsignificant association with poorer survival (HR = 1.30, 95% CI: 0.87–1.93, p = 0.203). In contrast, chRCC and pRCC exhibited minimal NOTCH1-ICD expression, with no observable impact on survival. NOTCH1-ICD was also detected in tumor endothelial cells, suggesting potential vascular mimicry. These findings indicate that NOTCH1-ICD may reflect tumor aggressiveness in ccRCC and could have implications for targeted therapy, but its independent prognostic value requires validation in larger cohorts.

## Introduction

The NOTCH1 signaling pathway governs key cellular processes, including proliferation, differentiation, and apoptosis. Upon ligand binding, the NOTCH1 receptor undergoes proteolytic cleavage, resulting in the release of the intracellular domain (NOTCH1-ICD), which translocates to the nucleus and activates downstream transcription.^1,2^ NOTCH1-ICD is thus a direct marker of pathway activation. Dysregulation of NOTCH signaling has been implicated in various malignancies, including renal cell carcinoma (RCC), a heterogeneous group of tumors accounting for 2–3% of adult cancers.^3,4^ Among RCC subtypes, clear cell RCC (ccRCC) is the most common and clinically aggressive. Papillary RCC (pRCC) and chromophobe RCC (chRCC) are generally less aggressive but biologically distinct.^5,6^ Although NOTCH pathway components have been studied in RCC, the specific expression pattern and prognostic significance of NOTCH1-ICD across histological subtypes remain poorly defined. Previously, our group identified a NOTCH1 mutation (p.L1575P_c4724T>C) in metastatic ccRCC that conferred sensitivity to the NOTCH1-ICD inhibitor CB103. In that case, immunohistochemistry (IHC) demonstrated predominant nuclear NOTCH1-ICD staining in tumor cells, although not all cancer cells were positive.^7^ Given that this alteration was identified in metastatic disease, we hypothesized that activation of the NOTCH1 pathway might be associated with more aggressive tumor behavior. Building on this observation, we evaluated NOTCH1-ICD expression by IHC in RCC and analyzed its association with clinicopathological characteristics and patient outcomes.

## Materials and Methods

Ethical approval was obtained from the Ethics Committee of the Medical Faculty, University of Belgrade, Serbia (approval number: 29/XII-2). This retrospective study included 101 patients who underwent nephrectomy for RCC between 2013 and 2016 at the University Clinical Center of Serbia. The study size was determined by the number of eligible cases with available tissue and follow-up data during the study period. Tumors were reclassified according to the 5^th^ edition WHO classification and staged per the 8^th^ edition AJCC criteria.^6–8^

Tissue microarrays (TMAs) were manually constructed from representative tumor areas. Three tumor cores per case were selected for TMA construction, representing both high- and low-grade areas to account for intratumoral heterogeneity.

IHC staining was performed using a rabbit monoclonal anti–NOTCH1-ICD antibody (clone D3B8, Cell Signaling Technology, dilution 1:100). NOTCH1-ICD expression was evaluated by IHC, with only nuclear staining considered positive. Each core was scored semiquantitatively: 0 (<1%), 1 (1–5%), 2 (5–10%), 3 (10–15%), and 4 (>15%). Expression variability between the three TMA cores was generally limited, with differences between cores typically not exceeding one scoring category. The overall score per case was derived by integrating the scores from all three cores. Scoring was performed independently by two pathologists (J.F. and S.R.Š.), with discrepancies resolved by consensus. Follow-up data were available for all patients and defined as the period from surgery until death or censoring in January 2024; cases with missing clinicopathological variables were excluded from the respective analyses.

Statistical analysis was conducted using SPSS v26. Categorical variables were compared using Chi-square or Fisher’s exact test. Continuous variables were analyzed using Mann–Whitney U or Kruskal–Wallis tests. Spearman correlation assessed variable associations. Cancer-specific survival (CSS) was analyzed by Kaplan–Meier with log-rank testing. Both univariate and multivariate analyses were performed, with multivariate analysis conducted using Cox proportional hazards regression to evaluate the independent prognostic significance of NOTCH1 expression, tumor grade, stage, tumor size, age, and sex. Cases with missing data were excluded from the respective analyses. Statistical significance was set at P < 0.05.

## Results

**Figure 1:**
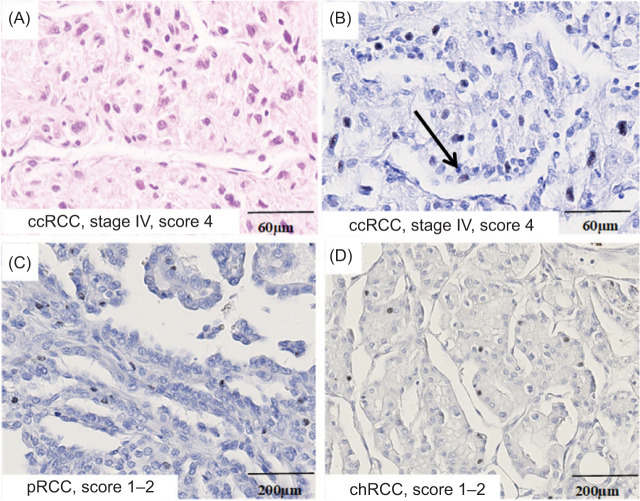
Representative microphotographs of heterogeneous NOTCH1-ICD expression in RCC subtypes showing (A) ccRCC grade and stage IV and (B) strong nuclear staining of NOTCH1-ICD in more than 15% of tumor cells in the same case of ccRCC, as well as in endothelial cells (arrow), (C) a lower percentage of NOTCH1-ICD positive cells in pRCC, and (D) chRCC, where no case showed more than 10% NOTCH1-ICD positive cells. Microscope magnification: 20×, 10×. RCC – Renal Cell Carcinoma; ccRCC – Clear Cell Renal Cell Carcinoma; pRCC – Papillary Renal Cell Carcinoma; chRCC – Chromophobe Renal Cell Carcinoma.

**Figure 2:**
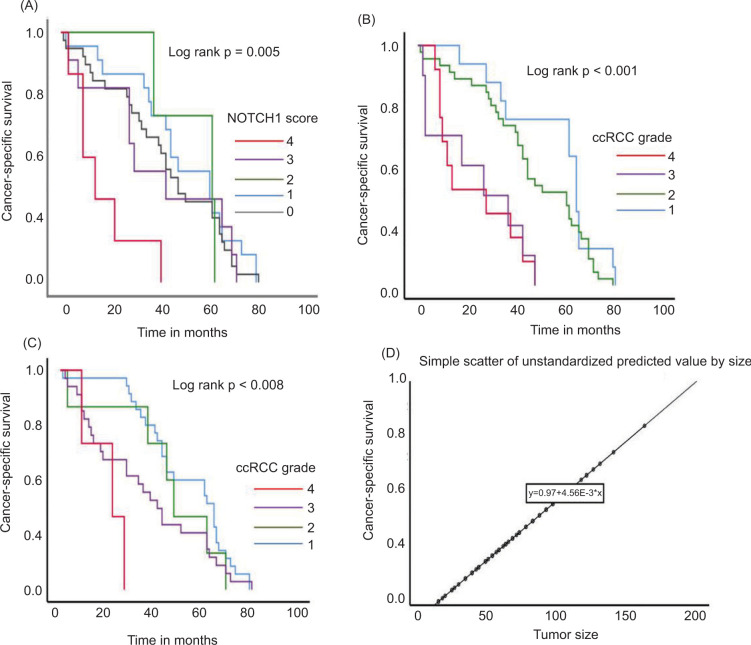
Survival analysis based on NOTCH1 score, tumor Grade, stage, and size in ccRCC revealed that (A) patients with higher NOTCH1 scores had significantly shorter CSS (P = 0.005), while in (B) a similar trend was observed, where a higher tumor grade (grade 4) was strongly associated with worse survival outcomes (P < 0.001). (C) Patients with more advanced tumor stage (stage 4) had significantly poorer prognoses compared to those with earlier-stage tumors (*P* < 0.008). (D) A positive correlation was observed between tumor size and the predicted NOTCH1 expression, suggesting that larger tumors tend to have higher NOTCH1 expression levels. The regression equation (y = 0.97 + 4.56E-3 * ×) further supports this association. RCC – Renal Cell Carcinoma.

As shown in Table 1, the cohort included 69 ccRCC, 15 pRCC, and 17 chRCC cases. For the whole cohort, the median age was 62 years, and 67.3% were male. Median follow-up was 43 months. CSS was the longest in pRCC (median 51 months), followed by chRCC (47 months), and the shortest in patients with ccRCC (41 months). Regarding tumor grade, most tumors were grade II, particularly in ccRCC (53.6%) and pRCC (53.3%). Higher grades (III and IV) were less common, while the grade was not defined for chRCC due to the lack of an appropriate grading system. The most common tumor stage was pT1, especially in chRCC and ccRCC, while advanced stage (pT4) was rare and seen only in ccRCC and pRCC. Tumor tissue revealed nuclear NOTCH1-ICD expression in cancer cells (Figure 1A–D). Interestingly, NOTCH1-ICD expression was also observed in tumor endothelial cells (Figure 1B). When analyzed according to NOTCH1-ICD expression levels, higher scores were predominantly observed in ccRCC, whereas pRCC and chRCC showed mostly low or absent expression. High NOTCH1-ICD expression was more frequent in high-grade and advanced-stage ccRCC tumors, while no significant association with patient age or sex was observed. A weak positive correlation was found between tumor size and NOTCH1-ICD score. The patients with ccRCC score 4 tumors had markedly poorer survival: 83.3% died during follow-up, and Kaplan–Meier analysis confirmed significantly shorter CSS in this group (log-rank *P* = 0.005) (Figure 2A). As expected, a greater number of patients with lower grade and tumor stage had better survival compared to their counterparts (log-rank *P* < 0.001 and *P* < 0.008, respectively) (Figures 2B–2C). A positive linear correlation between tumor size and NOTCH1-ICD expression suggests that larger tumors tend to exhibit slightly higher levels of predicted NOTCH1-ICD expression (Figure 2D).

**Table 1:** Distribution of clinicopathological variables according to NOTCH1-ICD expression scores (0–4) in clear cell, papillary, and chromophobe renal cell carcinoma.

Tumor Type	Variable	Score 0	Score 1	Score 2	Score 3	Score 4	p-value
**ccRCC (N=69**)	NOTCH1 score N (%)	18 (26.1%)	32 (46.4%)	10 (14.5%)	3 (4.3%)	6 (8.7%)	0.554
	Age (±SD)	60±8.5	62±10	64±6	65±6	63±8	0.306
	Gender (F/M)	5/13	11/21	3/7	1/2	3/3	0.900
	Tumor size (mm)	58±29	63±32	69±22	82±12	85±43	0.046*
	Grade	I: 7 (38.9%) II: 7 (38.9%) III: 0 (0.0%) IV: 4 (22.2%)	I: 5 (15.6%) II: 21 (65.6%) III: 4 (12.5%) IV: 2 (6.2%)	I: 2 (20.0%) II: 4 (40.0%) III: 3 (30.0%) IV: 1 (10.0%)	I: 0 (0.0%) II: 2 (66.7%) III: 1 (33.3%) IV: 0 (0.0%)	I: 0 (0.0%) II: 3 (50.0%) III: 2 (33.3%) IV: 1 (16.7%)	0.154
	Stage	pT1: 10 (55.6%) pT2: 3 (16.7%) pT3: 5 (27.8%) pT4: 0 (0.0%)	pT1: 13 (40.6%) pT2: 2 (6.2%) pT3: 16 (50.0%) pT4: 1 (3.1%)	pT1: 4 (40.0%)pT2: 2 (20.0%)pT3: 4 (40.0%)pT4: 0 (0.0%)	pT1: 0 (0.0%) pT2: 1 (33.3%)pT3: 2 (66.7%)pT4: 0 (0.0%)	pT1: 1 (16.7%)pT2: 0 (0.0%)pT3: 3 (50.0%)pT4: 2 (33.3%)	0.043*
	Outcome (died/total)	2/18 (11.1%)	9/32 (28.1%)	3/10 (30.0%)	0/3 (0.0%)	5/6 (83.3%)	0.012*
**pRCC (N=15)**	NOTCH1 score N (%)	3 (20.0%)		7 (46.7%)	1 (6.7%)	2 (13.3%)	2 (13.3%)
	Age (±SD)	72±11	61±6	52±20	52±20	64±1	0.236
	Gender (F/M)	0/3	2/5	0/1	1/1	0/2	0.567
	Tumor size (mm)	43±30	57±21	57±21	80±21	30±28	0.941
	Grade	I: 1 (50.0%) II: 1 (50.0%) III: 0 (0.0%) IV: 0 (0.0%)	I: 0 (0.0%) II: 3 (42.9%) III: 3 (42.9%) IV: 1 (14.3%)	I: 0 (0.0%) II: 2 (100.0%) III: 0 (0.0%) IV: 0 (0.0%)	I: 0 (0.0%) II: 2 (100.0%) III: 0 (0.0%) IV: 0 (0.0%)	I: 0 (0.0%) II: 1 (50.0%) III: 0 (0.0%) IV: 1 (50.0%)	0.378
	Stage	pT1: 1 (33.3%) pT2: 0 (0.0%) pT3: 2 (66.7%) pT4: 0 (0.0%)	pT1: 6 (75.0%) pT2: 0 (0.0%) pT3: 1 (12.5%) pT4: 1 (12.5%)	pT1: 0 pT2: 0 pT3: 0 pT4: 0	pT1: 0 (0.0%) pT2: 1 (50.0%)pT3: 1 (50.0%)pT4: 0 (0.0%)	pT1: 0 (0.0%)pT2: 1 (50.0%)pT3: 1 (50.0%)pT4: 0 (0.0%)	0.237
	Outcome (died/total)	0/3 (0.0%)	2/7 (28.6%)	0/1 (0.0%)	0/2 (0.0%)	0/2 (0.0%)	0.731
**chRCC (N=17)**	**NOTCH1 score N (%)**	**4 (23.5%)**	**9 (52.9%)**	**4 (23.5%)**	**0 (0.0%)**	**0 (0.0%)**	
	Age (±SD)	67±19	56±13	62±19	/	/	0.516
	Gender (F/M)	4/0	3/6	0/3	/	/	0.020*
	Tumor size (mm)	88±31	58±34	67±30	/	/	0.229
	Grade	–	–	–	/	/	—
	Stage	pT1: 1 (25.0%) pT2: 1 (25.0%) pT3: 2 (50.0%) pT4: 0 (0.0%)	pT1: 4 (57.1%) pT2: 0 (0.0%) pT3: 3 (42.9%) pT4: 0 (0.0%)	pT1: 2 (33.3%)pT2: 4 (66.7%)pT3: 0 (0.0%)pT4: 0 (0.0%)	0/0 (0.0%)	0/0 (0.0%)	0.309
	Outcome (died/total)	0/4 (0.0%)	1/9 (11.1%)	1/4 (25.0%)	0/0 (0.0%)	0/0 (0.0%)	0.494

RCC – Renal Cell Carcinoma; ccRCC – Clear Cell RCC; pRCC – Papillary RCC; chRCC – Chromophobe RCC; p – Statistical value; mm – Millimeter; *statistically significant value (p<0,05).

**Figure 3:**
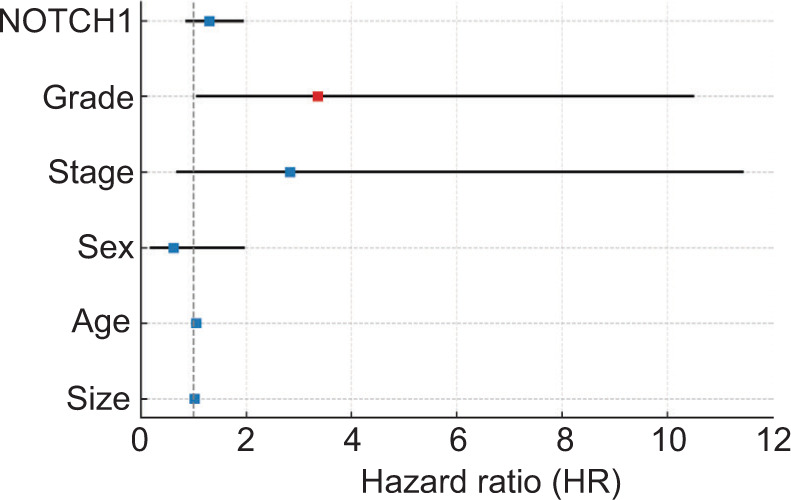
Forest plot of multivariate predictors of overall survival. Forest plot presenting the multivariate Cox regression analysis of overall survival, including tumor grade, NOTCH1-ICD expression, tumor stage, sex, age, and tumor size. Tumor grade is the only variable that remains an independent predictor, showing a clearly elevated hazard ratio. NOTCH1-ICD does not reach statistical significance as an independent factor, yet it still shows a consistent trend toward increased risk, indicating that its effect is present but not strong enough in this model. All other variables display hazard ratios crossing the reference line (HR = 1), suggesting no independent prognostic contribution in this cohort. NOTCH1-ICD-NOTCH1 intracellular domain; HR – Hazard ratio.

In the multivariate Cox regression analysis, tumor grade emerged as the only independent predictor of overall survival, with higher-grade tumors associated with a significantly increased risk of death (HR = 3.36, 95% CI: 1.07–10.49, p = 0.037). Patients with high NOTCH1 expression had numerically poorer survival; however, this association did not reach statistical significance (HR = 1.30, 95% CI: 0.87–1.93, P = 0.203). Other clinical and pathological variables, including tumor stage, sex, age, and tumor size, were not independently associated with survival in this cohort (Figure 3).

In pRCC, expression was generally low, without significant association with tumor size, grade, stage, or outcome. Similarly, chRCC cases exhibited low scores, with no patients scoring above 2. All chRCC patients with score 0 were female (*P* = 0.020), although this had no survival impact. NOTCH1-ICD expression was not significantly correlated with tumor size or pT stage in chRCC or pRCC.

## Discussion

NOTCH1 signaling is known to regulate cell differentiation, proliferation, and apoptosis, and its dysregulation contributes to tumor progression in several cancers, including RCC. Activation of NOTCH1 leads to cleavage of its intracellular domain, which translocates to the nucleus and modulates transcription of target genes involved in epithelial–mesenchymal transition (EMT), angiogenesis, and stemness. In ccRCC, NOTCH1 overexpression has been linked to increased vascular endothelial growth factor production and enhanced endothelial cell activation, supporting tumor vascularization. In the present study, increased NOTCH1-ICD expression was associated with shorter CSS in patients with ccRCC, supporting its link to more aggressive tumor behavior. The nuclear localization of NOTCH1-ICD observed in our cases is consistent with its transcriptionally active state and may underline the association between high NOTCH1-ICD scores and aggressive morphological features. These findings suggest that NOTCH1 activation could contribute to ccRCC progression through EMT-related and angiogenic mechanisms, although functional validation is needed. In ccRCC, increasing NOTCH1-ICD expression appears to mark biologically aggressive tumors, as indicated by larger size, higher stage, and shorter survival. In the multivariate Cox regression model, NOTCH1-ICD did not remain an independent prognostic factor once adjusted for tumor grade, stage, and other clinicopathological variables. This indicates that the prognostic relevance of NOTCH1-ICD expression is largely dependent on its association with adverse histopathological features, particularly higher tumor grade. Prior studies have evaluated total NOTCH1 protein or mRNA levels in RCC.^1–9,11^ However, NOTCH1-ICD provides a more direct measure of pathway activation. Functional studies have shown that NOTCH1 promotes tumor angiogenesis independently of VHL/HIF signaling.^6^ Our finding of endothelial NOTCH1-ICD expression, possibly due to vascular mimicry as previously evidenced by our research team,^7^ supports increased angiogenesis driven by direct tumor trans-differentiation into tumor endothelial-like cells in ccRCCs, even when only a minority of tumor cells express NOTCH1-ICD. Interestingly, the score 4 group in ccRCC, while numerically small (n = 6), showed distinct clinicopathological features and the worst survival, suggesting that high NOTCH1-ICD expression may define a high-risk subgroup. This indicates that the apparent effect of NOTCH1 may, at least in part, reflect tumor aggressiveness rather than a fully independent prognostic role. Nonetheless, the identification of high NOTCH1-ICD expression in aggressive ccRCC tumors raises potential therapeutic implications. These high-risk tumors could potentially benefit from targeted NOTCH inhibition using agents such as CB103, as suggested by preclinical and early clinical studies.^7–11^ Thus, while NOTCH1-ICD may not yet be confirmed as an independent prognostic biomarker, it could serve as a marker for tumors that are biologically aggressive and potentially responsive to targeted therapy.

The limited expression of NOTCH1-ICD in chRCC and pRCC highlights the divergent biology among RCC subtypes and underscores the need for subtype-specific biomarker strategies. Previous studies have reported differential expression of NOTCH pathway components across histological variants.^5–12,13^ Our findings refine these observations by demonstrating that NOTCH1-ICD expression correlates specifically with aggressive features in ccRCC but not in other subtypes. We recognize the potential impact of intratumor heterogeneity, particularly in ccRCC, which may influence biomarker assessment.^14^ To address this, three tumor cores were selected for each case in TMA construction, aiming to represent both high- and low-grade regions when present. The overall NOTCH1-ICD score was derived by integrating all three cores, providing a more representative assessment of expression across the tumor.

This study has several limitations. Being a single-center, retrospective analysis with relatively small numbers in some subgroups, the precision and generalizability of the findings may be limited. Semiquantitative scoring, although practical, may miss subtle expression differences; however, the use of a validated antibody and controlled TMA-based assessment ensured internal consistency. These results should be validated in larger, multicenter cohorts to confirm the prognostic and therapeutic relevance of NOTCH1-ICD in ccRCC.

## Conclusion

In conclusion, tumor grade remains the primary independent prognostic factor in ccRCC. Higher NOTCH1-ICD expression was observed in a subgroup of tumors with aggressive clinicopathological features and poorer survival; however, this association did not reach statistical significance. Although NOTCH1-ICD was not independently predictive in this cohort, its association with high-grade tumors suggests potential clinical relevance, particularly as a candidate biomarker for targeted therapy with NOTCH inhibitors such as CB103. These findings warrant further validation in larger, multicenter cohorts to better define the prognostic significance of NOTCH1-ICD and to explore its potential as a biomarker for targeted therapeutic strategies in ccRCC.
